# Folliculogenesis-Associated Genes Expression in Human Vitrified Ovarian Tissue after Xenotransplantation in γ-Irradiated Mice 

**DOI:** 10.22074/cellj.2020.6553

**Published:** 2019-12-15

**Authors:** Zahra Shams Mofarahe, Marefat Ghaffari Novin, Mojdeh Salehnia

**Affiliations:** 1 Department of Biology and Anatomical Sciences, School of Medicine, Shahid Beheshti University of Medical Sciences, Tehran, Iran; 2 Department of Anatomical Sciences, Faculty of Medicine, Tarbiat Modares University, Tehran, Iran

**Keywords:** Gene Expression, Ovarian Tissue, Vitrification, Xenotransplantation

## Abstract

**Objective:**

Autograft transplantation of vitrified cortical ovarian tissue is an acceptable clinical technique for fertility preservation in women. Xenograft transplantation into animal models could be useful for evaluating the safety of human vitrified ovarian tissue. This study targeted to evaluate impact of vitrification on expression of the genes associated with folliculogenesis after xenograft transplantation of human vitrified ovarian tissue to γ-irradiated mice.

**Materials and Methods:**

In this experimental study, ovarian biopsies were gathered from six transsexual persons. The cortical section of ovarian biopsies was separated and chopped into small pieces. These pieces were randomly divided into vitrified and non-vitrified groups. In each group some pieces were considered as non-transplanted tissues and the others were transplanted to γ-irradiated female National Medical Research Institute (NMRI) mice. Before and after two weeks of xenograft transplantation, histological assessment and evaluation of the expression of folliculogenesis- associated genes (*FIGLA, GDF-9, KL* and *FSHR*) were performed in both vitrified and non-vitrified groups.

**Results:**

Percentage of the normal follicles and expression of the all examined genes from transplanted and non- transplanted tissue were similar in both vitrified and non-vitrified groups (P>0.05). After transplantation, the normal follicle rate was significantly decreased and among the folliculogenesis-associated genes, expression of *GDF-9* gene was significantly increased, rather than before transplantation in vitrified and non-vitrified tissues (P<0.05).

**Conclusion:**

The vitrification method using dimethyl solphoxide and ethylene glycol (EG) had no remarkable effect on the normal follicular rate and expression of folliculogenesis-associated genes after two weeks human ovarian tissue xenografting. In addition, transplantation process can cause a significant decrease in normal follicular rate and expression of *GDF-9* gene.

## Introduction

There are a large number of primordial follicles in human ovarian cortical tissue (1). These follicles are more impervious to cryoinjury effects because of their low metabolism and lack of zona and cortical granules (2). Therefore, ovarian tissue cryopreservation is an acceptable method for fertility preservation in women (3, 4). The possibility of harvesting ovarian tissue at any time of the menstrual cycle is one of hallmarks of fertility preservation through ovarian tissue cryopreservation, in comparison with the other methods. Hence, in cancerous patients with limited time for ovulation induction and egg collection or in pre-pubertal girls, ovarian tissue cryopreservation assumes as the only chance for fertility preservation (5). Recently, the simplicity, safety and cost effectiveness of vitrification made it more acceptable technique, although there are different cryopreservation methods (6-10).

Normal development of follicle needs expression of the particular genes involved in the folliculogenesis process (11, 12). Accordingly, it has been revealed that expression of *FIGLA* and *GDF-9* genes in oocyte of primordial and primary follicles respectively, in addition to expression of *KIT LIGAND (KL)* and *FSHR* genes in the granulosa cells of primordial and secondary follicles play an essential role in the follicular development (13-16). No significant alteration on the expression profile of these genes has been detected in human vitrified ovarian tissue immediately after warming or following two weeks of *in vitro* culture (17, 18).

Autograft transplantation of vitrified ovarian tissue was a successful clinical procedure because endocrine function as well as fertility was resumed in patients (19, 20). Contradictory findings have been reported regarding the alteration of oocyte and follicular cells gene expression in vitrified ovarian tissue after transplantation (21-23). In this context, xenograft transplantation into animal models proposed as a quality evaluation of human vitrified ovarian tissues before clinical usage (22).

Since there was a lack of study in expression of the genes involved in follicular development of human vitrified ovarian tissue after the transplantation, the goal of this study was to evaluate impact of vitrification on expression of genes involved in folliculogenesis after xenograft transplantation of human vitrified ovarian tissue to γ-irradiated mice.

## Materials and Methods

All reagents and materials were obtained from SigmaAlderich (Germany) except those mentioned.

### Ovarian tissue collection

This experimental study was endorsed by the Ethics Commission of faculty of Medicine of Shahid Beheshti University of Medical Science (Tehran, Iran) and informed consents were obtained for usage of human tissues (no.172). In this comparative research, ovarian tissue samples were obtained during sex reassignment surgery from six transsexual persons aged 20-30 years with informed consent. It should be noted that persons with a history of hormone administration were excluded. The specimens were promptly delivered to the laboratory in Leibovitz’sL-15 medium on ice, as described in the previous studies (17, 18).

### Preparation of human ovarian cortical tissue

The ovarian biopsies were transferred to fresh equilibrated Leibovitz’sL-15 medium. Then their cortical tissues were separated and chopped into small pieces (4×2×1 mm) under a sterile condition.

The retrieved ovarian tissue pieces were accidentally separated into vitrified and non-vitrified groups (n=100 pieces in total). In both non-vitrified and vitrified groups, 50 pieces (from at least five women) were regarded as nontransplanted tissues. Among these tissues, 30 pieces were fixed in Bouin’s solution for histological assessment and 20 pieces were kept at -80˚C for later molecular evaluation. In both non-vitrified and vitrified groups, the remaining pieces (n=50 pieces from at least five women) were transplanted to 25 γ-irradiated immunosuppressed female mice for two weeks. From these transplanted tissues, 30 pieces were fixed in Bouin’s solution for histological assessment and 20 pieces were kept at -80˚C for later molecular evaluation.

### Vitrification and warming procedures

The ovarian tissue pieces were vitrified by the described procedure of Kagawa et al. (24) with some modification. In summary, the pieces were initially washed out in Hanks’ balanced salt solution (HBSS with HEPES) complemented with 20% human serum albumin (HAS), then immersed in equilibration solution containing HBSS, 7.5% ethylene glycol (EG) and 7.5% dimethyl sulphoxide (DMSO) for 25 minutes. Next, the tissue pieces were placed into the vitrification solution (20% EG, 20% DMSO and 0.5 M sucrose) for 15 minutes. Finally, the tissue pieces were moved into cryovials containing 100 μl vitrification solution set on nitrogen vapor for 30 seconds and then kept in liquid nitrogen for a week.

The pieces were melted by plunging the vials into water bath at 37˚C. Then, they were transferred into HBSS containing 1 M sucrose in for 3 minutes at 37˚C, and incubated in 0.5 M sucrose for 5 minutes at room temperature. Finally, the pieces were equilibrated in α-MEM medium for two hours.

### Providing γ-irradiated mice and transplantation of the human ovarian tissue

Female NMRI mice were obtained from Tarbiat Modares University animal house (Tehran, Iran), aged between 8 and 10 weeks, and used in this study. The mice were synchronized and phase of the mice estrous cycle was confirmed by vaginal cytology. The vaginal smear viewed under a light microscope at the ×400 magnification and they were considered for human ovarian transplantation at postures phase. All experimental procedures were accepted by Animal Research Committee of Shahid Beheshti University. In order to suppress the immune system for preventing rejection of the transplanted tissue, the mice (n=25) were irradiated with 7.5 Gy single dose Gama irradiation for 6 minutes (25).

Transplantation of cortical ovarian tissue was preformed 72 hours after irradiation. Intra-peritoneal injection of a combination of ketamine 10 % (75 mg/ kg body weight) and xylazine 2 % (15 mg/kg) was administered for anesthesia. Two pieces of human ovarian tissue were subcutaneously transplanted into the back of each mouse. Then the mice were kept under aseptic situation with free availability to food and water. After 14 days of transplantation, the mice were euthanized by cervical dislocation and tissue fragments were retrieved for downstream experiments.

### Histological evaluation by hematoxylin and eosin

A total of 15 pieces in each group of the study were fixed in Bouin’s solution for 18 hours at room temperature. The samples were processed and embedded in paraffin wax, and they were subsequently serially sectioned at 5 μm thickness. Every tenth section of each piece was mounted on glass slides and colored with hematoxylin and eosin (H&E). Then, each section was inspected for determining the number of follicles, field by field under the ×100 magnification of the light microscope. In order to prevent the re-counting follicles, only follicles with a clear nucleus of oocyte were calculated. The follicles were classified as primordial, primary and secondary according to the previous classification (26). Primordial follicles had one layer of flattened follicular cells. Primary follicles had one layer of cuboidal follicular cells and secondary follicles had two or more layers of cuboidal granulosa cells. Atretic follicles had pyknotic oocyte nucleus, shrunken ooplasm or disorganized follicular cells.

### RNA extraction and cDNA synthesis for molecular evaluation

In order to evaluate expression of the some genes related to development of oocyte and follicular cells (including: *FIGLA, GDF-9, KL* and *FSHR* genes) total RNA was extracted from 40 non-transplanted and transplanted fragments in both vitrified and non-vitrified groups using TRIzol reagent (Invitrogen, USA) as indicated by the manufacturer’s directions. The RNA specimens were treated with DNase to eliminate any genomic DNA contamination only before proceeding with the cDNA synthesis. Then, RNA concentration was calculated by spectrophotometry. Finally, 1000 ng of the extracted RNA was used for cDNA synthesis by the commercial Kit (Thermo Scientific, EU), according to the indicated manufacturer’s directions. The cDNA synthesis reaction was carried out at 42˚C for 60 minutes, and the synthesized cDNA was kept at -20˚C.

### Real-time reverse transcriptase-polymerase chain reaction

The primers for real-time reverse transcriptasepolymerase chain reaction (RT-PCR) were formulated (Table 1) utilizing GenBank (http://www.ncbi.nlm.nih. gov) and Primer3 software, then synthesized by Generary Biotech Company (China).

RT-PCR was carried out by the Applied Biosystems (UK) real-time thermal cycler as indicated by QuantiTect SYBR Green RT-PCR Kit (Applied Biosystems, UK). The housekeeping gene, *β-ACTIN*, was considered as internal control. For each specimen, the house keeping gene and the target genes were amplified in the same round. One microliter of cDNA, 1 μl of the mixture of forward and reverse primers and 10 μl SYBR Green Master Mix were used per 20 μl of the reaction volume. After each PCR run, melt curve was analyzed to determine amplification specificity. Real-time heating condition included holding step at 95˚C for 5 minutes, cycling steps (35-40 cycles) at 95˚C for 15 seconds, 58˚C for 30 seconds and 72˚C for 15 seconds which was continued by a melt curve analysis at 95˚C for 15 seconds, 60˚C for 1 minutes and 95˚C for 15 seconds. Then the relative quantification of target genes was calculated by the Pfaffl formula (27). The real-time RT-PCR experiments were performed duplicate for each specimen in at least a three biological repeats.

### Statistical analysis

Statistical analysis was performed with the SPSS 19.0 software (SPSS Inc., Chicago, IL, USA). Quantitative variables were displayed as mean ± standard error (SE) and percentage. The follicular counting data and result of realtime RT-PCR were analyzed by paired-samples t test and bootstrap. P<0.05 were considered statistically significant.

## Results

### Histological examination of ovarian cortical tissue

**Fig 1:**
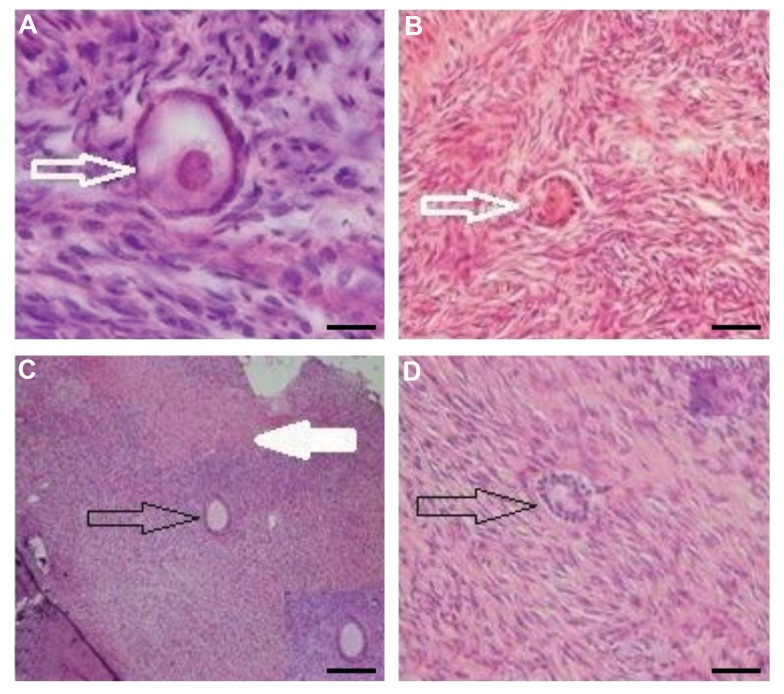
Hematoxylin and eosin staining of non-transplanted and transplanted human ovarian cortical sections in both vitrified and non-vitrified groups. The morphology of primordial and primary follicles was shown white arrow and the secondary follicles demonstrated black arrow. **A.** Vitrified nontransplanted tissues, **B.** Vitrified transplanted tissue, **C.** Non-vitrified non-transplanted group, and **D.** Non-vitrified and non-transplanted group. The atretic follicles were shown in fibrotic and ischemic areas of transplanted tissues (white filled arrow in B) (scale bar: 200 µm).

The morphology of non-transplanted and transplanted ovarian cortical tissues in both vitrified and non-vitrified groups was shown in Figure 1. In both vitrified and non-vitrified groups, the morphology of follicles and stromal cells was almost similar, before and after transplantation. The normal follicles had circular oocyte with intact granulosa cells. The atretic follicles were seen in fibrotic and ischemic areas of transplanted tissues. The stromal cells had also a normal appearance in all groups. After 14 days of ovarian xenograft, the growing follicles (secondary follicles) were seen in vitrified and non-vitrified tissues (Fig .1). The follicular integrity and stromal tissue structures of transplanted and non-transplanted tissues were similar in vitrified and non-vitrified groups.

**Table 1:** Specifications of the primers utilized for real-time reverse transcriptase-polymerase chain reaction (RT-PCR) assay


Target gene	Primer sequence	Accession number	Product size (bp)

*β-actin*	F: TCAGAGCAAGAGAGGCATCC	NM_001101.3	187
	R: GGTCATCTTCTCACGGTTGG		
*FIGLA*	F: TCGTCCACTGAAAACCTCCAG	NM_001004311.3	76
	R: TTCTTATCCGCTCACGCTCC		
*KL*	F: AATCCTCTCGTCAAAACTGAAGG	NM_000899.4	163
	R: CCATCTCGCTTATCCAACACTGA		
*GDF-9*	F: TCCACCCACACACCTGAAAT	NM_005260	147
	R: GCAGCAAAACCAAAGGAGGA		
*FSHR*	F: CTGGCAGAAGAGAATGAGTCC	NM_181446.2	157
	R: TGAGGATGTTGTACCCGATGATA		


### Percent of normal follicle in the non-transplanted ovarian tissues

A total of 350 follicles were counted in 30 pieces of non-vitrified and vitrified ovarian tissues. The percentages of morphologically normal follicles at different developmental stages in both groups are shown in Table 2. This rate was 86.5% in non-vitrified and 84.3% in vitrified tissues. In non-vitrified tissue, among normal follicles the proportion of primordial, primary and secondary follicles was 62.4%, 22.1% and 2%, respectively. In vitrified group, these percentages were 59%, 23.1% and 2.2%, respectively. There was no statistically significant difference in the percentage of normal follicles between these two groups (P>0.05).

### Percentage of normal follicle in the transplanted ovarian tissues

A total of 300 follicles were counted and analyzed in 30 transplanted ovarian cortical pieces in the non-vitrified and vitrified groups. Percentage of the morphologically normal follicles, at different developmental stages in both groups, are shown in Table 2. Proportion of normal follicle was 78.9% in non-vitrified and 75% in vitrified tissues. Among the normal follicles in non-vitrified and vitrified tissues, percentage of the primordial follicles was 14.4% and 15.8%, primary follicles was 55.1% and 52.5%, secondary follicles was 9.4% and 6.7%, respectively. There was no statistically significant difference between these two groups (P>0.05).

### Comparison of normal follicle percentage in the nontransplanted and transplanted ovarian tissues

In the both transplanted tissues, compared to the nontransplanted tissues, percentage of normal follicles was significantly decreased (P<0.05). Proportion of primordial follicles was significantly lower and percent of primary and secondary follicles was significantly higher in both transplanted tissues than their non-transplanted counterparts (P<0.05).

**Table 2:** Percentage of the normal follicles at different developmental stages before and after xenograft transplantation of human ovarian tissue in vitrified and non-vitrified groups


Group	Number of total follicles	Number of normal follicles	Number of primordial follicles	Number of primary follicles	Number of secondary follicles

Non-transplanted vitrified	155	131/155^*^^*^	135/131^*^^*^	57/131^*^^*^	9/131^*^^*^
		(84.51 ± 1.42)	(58.69 ± 2.61)	(23.24 ± 2.48)	(2.58 ± 0.54 )
Non-transplanted non-vitrified	195	169/195^*^	166/169^*^	68/169^*^	13/169^*^
		(86.66 ± 2.11)	(63.58 ± 5)	21.02 ± 4.68	(2 ± 1.71)
Transplanted vitrified	120	90/120^*^^*^	19/90^*^^*^	63/90^*^^*^	8/90^*^^*^
		(75 ± 3)	(15.83 ± 1.21)	(52.50 ± 3)	(6.66 ± 1 )
Transplanted non-vitrified	180	142/180^*^	26/142^*^	99/142^*^	17/142^*^
		(78.88 ± 1.33)	(14.44 ± 1.58)	(54.99 ± 2.12)	(9.44 ± 1.50)


Data were presented as mean (%) ± SE. There was no significant difference between the vitrified and non-vitrified groups before and after transplantation in all columns (P>0.05). There were significant differences between transplanted and non-transplanted groups in vitrified (*) and non-vitrified group (**), P<0.05.

### Expression of folliculogenesis-associated genes in the non-transplanted ovarian tissues

**Fig 2:**
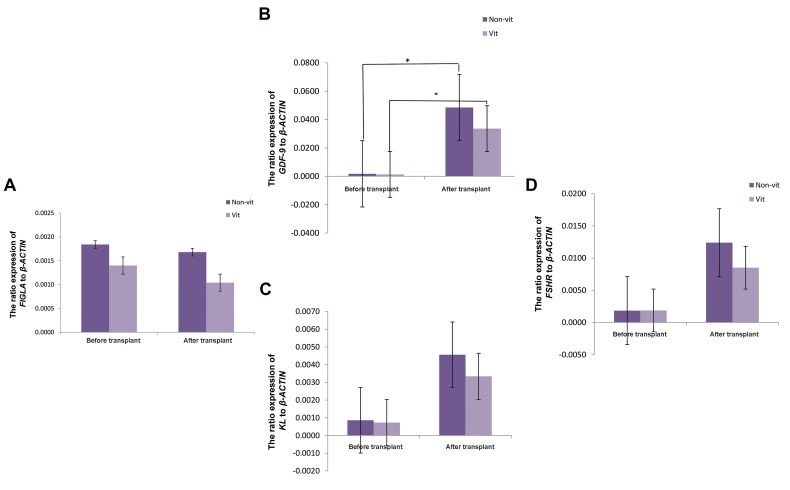
Folliculogenesis-associated genes expression to *β-ACTIN* in nontransplanted and transplanted human ovarian cortical tissues of vitrified and non-vitrified groups. **A.** *FIGLA*, **B.** *GDF-9*, **C.** *KIT LIGAND*, and **D.** *FSHR* genes expression to *β-ACTIN* in the non-transplanted and transplanted tissues in non-vitrified and vitrified groups are shown. There was no statistically significant difference between non-vitrified and vitrified groups. *; The expression of *GDF-9* gene was significantly increased in transplanted tissues in comparison with non-transplantation tissues.

The ratio expression of *FIGLA, GDF-9, KL* and *FSHR* genes to *β-ACTIN* gene in non-vitrified group before transplantation were 18.4×10^-4^, 17.3×10^-4^, 8.6×10^-4^ and 18.4×10^-4^ while in non-transplanted-vitrified group they were respectively 14×10^-4^, 13×10^-4^, 7.3×10^-4^ and 18.7×10^-4^ (Fig .2). There was no statistically significant difference between expression of the all examined genes in both non-transplanted tissues (P>0.05).

### Expression of folliculogenesis-associated genes in the transplanted ovarian tissues

The ratio expression of *FIGLA, GDF-9, KL* and *FSHR* genes to *β-ACTIN* gene in non-vitrified group after transplantation were respectively 16.8×10_-4_, 484.7×10_-4_, 45.6×10_-4_, 123.9×10_-4_ while they were respectively 10.4×10_-4_, 335.8×10_-4_, 33.4×10_-4_ and 85.1×10_-4_ in the vitrified group (Fig .2). There was no statistically significant difference between expression of the all examined genes in the both transplanted-vitrified and non-vitrified groups (P>0.05).

### Comparison of folliculogenesis-associated gene expressions in non-transplanted and transplanted ovarian tissues

In the transplanted tissues in comparison with nontransplanted tissues, **FIGLA, KL** and **FSHR** gene expression levels were similar in both vitrified and nonvitrified groups (P0.05). But, **GDF-9** gene expression was significantly increased in the transplanted tissue of the vitrified and non-vitrified groups (P<0.05).

## Discussion

In this study, we showed that rate of normal follicles at different developmental stages in the cortical ovarian tissue was similar after 14 days of xenograft transplantation in both vitrified and nonvitrified transplanted groups. It seems that method of vitrification had no delayed deleterious effects on normal follicles rate in human ovarian tissue, and primordial and primary follicles were able to resume growth and development after vitrification and transplantation. Therefore, this method is useful for preserving human ovarian cortical tissue. Application of EG and DMSO as penetrable and sucrose as nonpenetrable cryoprotectant in the vitrification solution, by reducing tissue damage, had a good effect on preserving ovarian cortical tissue.

Consistent to our findings, David and colleagues showed that integrity of follicles in cryopreserved human ovarian tissue was preserved well after three weeks xenotransplantation (28). Moreover, Nisdle reported that after three weeks xenograft transplantation of human ovarian cortical tissue, there was no significant difference between normal follicle population in the both vitrified and nonvitrified transplanted tissue (29). In contrast with our data, Jafarabadi and colleagues showed that xenotransplantation of vitrified tissue resulted in a much more reduction of the normal follicles in comparison with non-vitrified one. They suggested that vitrification procedure might have harmful effects on the viability and morphology of follicles after transplantation (22). This discrepancy could be due to differences in the vitrification techniques, transplantation site and duration of transplantation.

The success of xenograft transplantation depends on many factors including the vitrification technique, composition of vitrification solution, type of species and transplantation site (30).

According to our obtained data transplantation of vitrified and non-vitrified human ovarian tissues resulted in a significant reduction in the rate of normal follicle apart from vitrification procedure. Moreover, fibrotic areas were observed in the transplanted tissue in both groups. Decreased rate of the normal follicles may be related to occurrence of ischemia during tissue transplantation. Hence, the follicle normality may be more sensitive to the transplantation procedure on its own than vitrification. The revascularization of transplanted tissue takes several days and during this period ischemia has occurred. This ischemia reduced number of follicles and subsequently diminished lifetime of the transplanted tissue (31). One of the most important factors in successful transplantation is rapid re-establishment of blood flow which is essential for survival of ovarian follicles in the transplanted tissue (32). Amorim et al. (7) have reported considerable follicular survival after transplantation of human ovarian cortical tissue to kidney capsule of immunosuppressed mice. In contrast to the above report, which preformed transplantation with a suitable vascular bed for transplanted ovarian tissues, herein, the degeneration of follicles due to ischemia could be attributed to inappropriate vascular bed support. Moreover, Dath et al. (33) reported that intramuscular transplantation of ovarian tissue associated with better survival of ovarian follicles because of a suitable blood supply. Hence, to improve the follicles viability, transplantation site with suitable blood vessels should be selected.

In this study, significant reduction of normal follicle rate and number of primordial follicles were noticed; however, the number of growing follicles (primary and secondary) was significantly increased at the end of transplantation. This may represent the progress of follicular development in transplanted vitrified and non-vitrified tissues in spite of non-suitable vascular bed. Accordingly, we observed similar gene expression levels of *FIGLA, GDF-9, KL* and *FSHR* in the vitrified and non-vitrified transplanted tissues. It seems that vitrification did not have any significant effect on mRNA level and ovarian follicles had retained their ability to express developmental genes even after transplantation. Compatible to our data, Jafarabadi and colleagues showed that anti-apoptotic gene expressions were similar in both vitrified and non-vitrified human ovarian tissue after one month transplantation (22). It has also been revealed that expression of *CKIT, KL* and *GDF-9* genes in human cryopreserved and fresh ovarian cortical tissue was similar after three weeks transplantation (28). As we observed, expression of the majority of target genes before and after transplantation in vitrified and nonvitrified tissue was similar and only expression of *GDF-9* gene in vitrified and non-vitrified tissue was significantly increased after transplantation. Increased number of the primary follicles may be related to the activation of primordial follicles following incidence of ischemia and hypoxia in the transplanted ovarian tissue (32). Considering that *GDF-9* gene are expressed in growing ovarian follicles (primary and secondary) (9), its increased expression could be because of elevated number of growing follicles after transplantation. This activation may result in loss of follicular reservoir in transplanted ovarian tissue that subsequently reduces lifespan of the transplanted tissue (32).

## Conclusion

The vitrification method using DMSO and EG had no harmful effect on the follicular development and expression of genes related to folliculogenesis after xenografting human ovarian tissue. Moreover, the process of transplantation can cause a significant decrease in normal follicular rate and expression of *GDF-9* gene.
